# Supplementary material for: Characterization of Two Aggressive PepMV Isolates Useful in Breeding Programs
Source: Viruses. 2023 Nov 8;15(11):2230. doi: 10.3390/v15112230 (PMC10674935; doi:10.3390/v15112230)
Supplement: Supplementary file 1 [file viruses-15-02230-s001.zip › viruses-2682353-supplementary/viruses-2682353-supplementary.pdf]

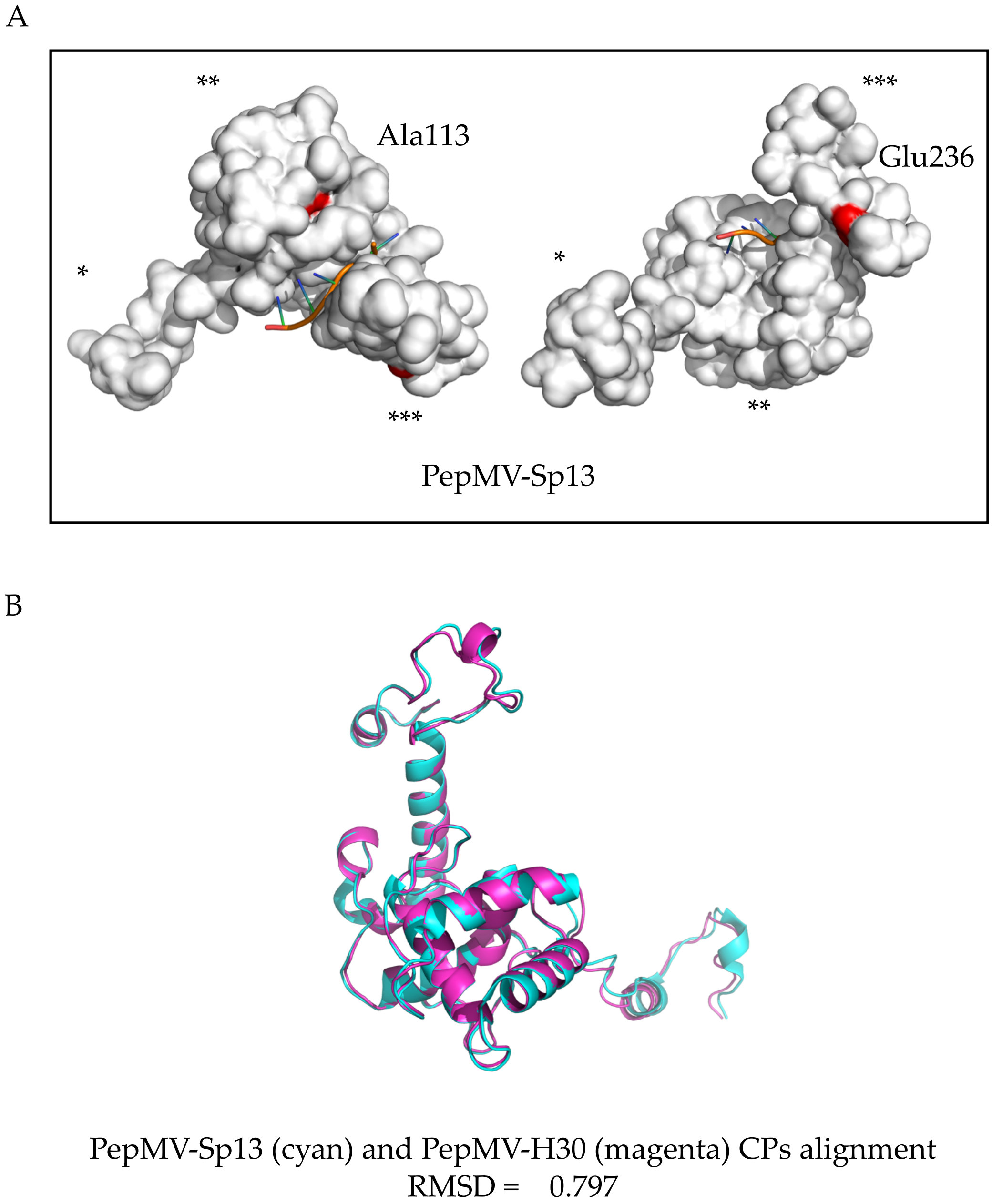

Figure S1. Position of residues Ala113 and Glu236 in the PepMV-Sp13 CP and alignment of the atomic models of the PepMV-Sp13 and PepMV-H30 CPs. (A) Atomic model of the PepMV-Sp13 CP represented in solvent accessibility mode. The viral RNA is shown in orange and residues Ala113 and Glu236 for the PepMV-Sp13 CP are colored in red. One, two or three asterisks indicates the N-terminal arm, the core region or the C-terminal extension of the CP. **(B)** Alignment of the PepMV-Sp13 (cyan) and PepMV-H30 (magenta) CPs. The root-mean-square deviation (RMSD) of the alignment is indicated in the figure.

Table S1: List of primers used for the sequencing of PepMV-H30 and PepMV-KLP2 isolates.

Primer	Sequence	Target isolate
CE-126	5'-GACTTTCATCTCTCTGACAGA-3'	PepMV-H30
CE-200	5'-GCCACCTCGGTTACATTGAAGC-3'	PepMV-H30 and PepMV-KLP2
CE-250	5'-CATGACTTCAAATTGAAGC-3'	PepMV-H30 and PepMV-KLP2
CE-290	5'-GTGGTGTCCAACAATTTCGAG-3'	PepMV-H30 and PepMV-KLP2
CE-301	5'-GAAAGAACTTCATATATACGC-3'	PepMV-H30
CE-306	5′-CGACCGACGCGTAACTTCTCCCCTTGGAACG-3′	PepMV-H30
CE-411	5′-CCTTCAGAACTCATAGATTTG-3′	PepMV-H30 and PepMV-KLP2
CE-412	5′-GGAGCTGTATTGGGATTTGAGAAGTC-3′	PepMV-H30
CE-432	5′-CCCAGCATTGCCACACAAG-3′	PepMV-H30
CE-434	5′-CTGGAATGCTTGGATCCCATT-3′	PepMV-KLP2
CE-435	5′-GCCATCTGGGCCGTATTG-3′	PepMV-KLP2
CE-501	5′-GAAAACTTCACCCGTTCCAAGTTATTTGTATAGTTCATC-3′	PepMV-H30
CE-503	5′-GAAAACTTAACCCGTTCCAAGTTAAAGTTCAGGGGGTG-3′	PepMV-H30
CE-504	5′-CACCCCCTGAACTTTAACTTGGAACGGGTTAAGTTTTC-3′	PepMV-H30
CE-505	5′-GTGCTTGCAACCATGTCCTTTT-3′	PepMV-H30
CE-508	5′-CCTTGCATTATCGATTGATGCA-3′	PepMV-H30
CE-1818	5'-GCAATCCAAACACATACTCATGC-3'	PepMV-H30 and PepMV-KLP2
CE-1819	5'-TGTGTAAGTTCATCAGGGTCATAC-3'	PepMV-KLP2
CE-1820	5'-AGTCATGTTGTATGGTACTATGGC-3'	PepMV-KLP2
CE-1906	5'-CCAGATGAAGCTGAACAAC-3'	PepMV-H30 and PepMV-KLP2
CE-1966	5'-ATGCCATGTCGACCC(T)30-3'	PepMV-H30 and PepMV-KLP2
CE-2158	5'-TCCTTCGGATGCCCAGGTCG-3'	PepMV-H30
CE-2159	5'-GACCTTCCTCTATATAAGG-3'	PepMV-H30 and PepMV-KLP2
CE-2173	5'-TCAATAAGGTGACACAAAGAGATATC-3'	PepMV-KLP2
